# Non-pharmacological postpartum interventions for management of maternal stress in postpartum period: A systematic review of clinical trials

**DOI:** 10.1097/MD.0000000000047814

**Published:** 2026-02-20

**Authors:** Khadije Rezaie Keikhaie, Maryam Nakhaee Moghadam, Shahla Faal Siahkal, Hayedeh Arbabi, Maryam Koochakzai

**Affiliations:** a Zabol Medicinal Plants Research Center, Zabol University of Medical Sciences, Zabol, Iran; b Department of Obstetrics and Gynecology, School of Medicine, Amir al Momenin Hospital, Zabol University of Medical Sciences, Zabol, Iran; c Department of Midwifery, Mara.C., Islamic Azad University, Marand, Iran; d Department of Midwifery, School of Nursing and Midwifery, Zabol University of Medical Sciences, Zabol, Iran.

**Keywords:** non-pharmacological intervention, postpartum period, stress, systematic review

## Abstract

**Background:**

In the crucial postpartum stage, mothers undergo substantial physiological and psychological shifts, with many experiencing stresses. Non-pharmacological interventions hold potential as a safe and effective way to manage postpartum stress compared to medication. The aim was to review non-pharmacological interventions for management of maternal stress in the postpartum period.

**Methods:**

In this systematic review, a comprehensive literature search was conducted on March 1, 2024 across databases including PubMed, Web of Science, and CINAHL, focusing on clinical trials published in English from inception until the end of 2023. Clinical trials utilizing interventions other than pharmacological agents for management of stress among postpartum women were included. The study outcome was postpartum stress measured by any standard scale. The risk of bias assessment was done using JADAD scale for randomized trials.

**Results:**

Electronic database searches initially yielded 9129 records, of which there remained 42 studies after removing duplicates and excluding records based on title and abstract screening. Finally, 15 studies that met the inclusion criteria were included in this review as a result of searching in databases and reviewing the reference list of related reviews. The most common non-pharmacologic interventions were: support group; home visiting program; mother-infant transaction program; listening to music; advanced practice nurses’ follow up through telephone calls; heart rate variability biofeedback; film addressing common stressors or a telephone support hotline; professionally-led telephone support or self-directed written support; home-based supportive-educational counseling; aerobic gymnastic exercise; telephone-based exercise or telephone-based wellness/support; home midwifery assistance; skin-to-skin contact; and social media-based parenting program. Despite the heterogeneity between the included studies, the majority demonstrated significant reduction in postpartum stress.

**Conclusion:**

Non-pharmacological interventions reduce postpartum stress, which may offer safe alternatives to pharmacological treatments, particularly for breastfeeding mothers.

## 1. Introduction

The postpartum period, also known as the puerperium, is a critical time for mothers, marked by significant physiological, psychological, and social changes.^[1,2]^ A significant proportion of mothers also experience heightened stress and anxiety, which can further exacerbate their mental health challenges.^[3]^ The postpartum period’s emotional and psychological demands necessitate effective management strategies to mitigate these risks and promote maternal and infant well-being.

Although the significance of managing postpartum stress is well-acknowledged, a dearth of comprehensive evaluations in the literature exists regarding non-pharmacological interventions. Previous studies have either focused on specific types of interventions or limited their scope to certain populations, thereby restricting the generalizability of their findings.^[4,5]^

Epidemiological data highlight the urgent need for effective interventions. For instance, studies have shown that untreated maternal stress can lead to long-term adverse outcomes including impaired cognitive and emotional development in children and increased risk of behavioral problems.^[6,7]^ These findings underscore the public health significance of addressing postpartum stress through effective and accessible interventions.

Non-pharmacological interventions, which encompass a variety of therapeutic approaches such as cognitive-behavioral therapy, mindfulness-based interventions, and support groups, have been increasingly recognized for their potential in managing stress during the postpartum period. These interventions offer a range of benefits including reduced symptoms of anxiety and depression, enhanced coping skills, and improved overall well-being without the side effects associated with pharmacological treatments.^[2,8]^ Furthermore, addressing postpartum stress through non-pharmacological approaches is particularly important given the potential risks associated with pharmacological treatments during breastfeeding. Many medications can pass into breast milk and may pose risks to the infant, making non-pharmacological interventions a safer and more appealing option for mothers. Therefore, the aim of this study was to review non-pharmacological interventions for managing maternal stress in postpartum period. This will offer a more complete understanding of the effectiveness of these interventions in managing maternal postpartum stress. On the other hand, by highlighting the benefits and effectiveness of non-pharmacological interventions, this review will promote their integration into standard postpartum care practices, ultimately improving outcomes for both mothers and infants.

## 2. Materials and methods

### 2.1. Data sources and searches

In this systematic review, we adhered to the preferred reporting items for systematic reviews and meta-analyses (PRISMA) statement.^[9]^ The PRISMA checklist is included as a supplementary material (Table S1, Supplemental Digital Content, https://links.lww.com/MD/R438).

In this study, a comprehensive literature search was conducted in the electronic databases including PubMed, Web of Science, and CINAHL. Initially, the search strategy was developed in the PubMed database using a combination of keywords and medical subject headings terms related to non-pharmacological interventions, postpartum stress, and management. Boolean operators (AND, OR, NOT) were utilized to refine the search. Then, it was modified for other databases as shown in Table S2, Supplemental Digital Content, https://links.lww.com/MD/R438.

The search was performed on March 1, 2024 without any date restriction to ensure a comprehensive collection of relevant literature. To ensure a thorough search, the reference lists of the relevant reviews were manually screened for additional studies.

### 2.2. Outcomes

Maternal stress in the postpartum period served as the primary outcome, measured through standardized scales.

### 2.3. Study selection

Two researchers (MK, KhRK) independently screened the title and abstract of the retrieved documents to determine their eligibility based on the following inclusion and exclusion criteria:

Inclusion criteria:
1. Study design: Randomized controlled trials (RCTs) or quasi-experimental studies.
2. Population: Women in the postpartum period with normal infants.
3. Intervention: Any non-pharmacological interventions applied to manage postpartum stress during the postpartum period.
4. Comparison: Regular/usual care in the postpartum period.
5. Outcome: Maternal postpartum stress based on any validated scale.
Exclusion criteria:
1. Studies without a control group.
2. Studies focusing on pharmacological treatments or invasive interventions.
3. Studies including mothers with severe psychiatric conditions such major depression, or infants born with congenital anomalies, neurological sequelae, hearing loss or chromosomal disorders.
4. Studies with parenting intervention and without reporting maternal stress.

The full-text of potentially eligible studies were then assessed independently by the same researchers. Any discrepancies between the researchers were resolved through discussion or consultation with a third reviewer (MNM).

### 2.4. Data extraction

Data were extracted using a standardized form that included information on study characteristics (e.g., author, year, country, study design, sample size, study population, type of intervention and its detail, comparison group, study outcome and its measurement, duration of intervention/follow-up, key findings and study limitations). Data extraction from the included studies was done by 2 authors (MK, ShFS), and any disagreements were resolved by discussing with a third author (MNM).

### 2.5. Risk of bias assessment

To assess the risk of bias in the included studies, we used the JADAD scale for reporting randomized controlled trials.^[10]^ The assessment was done by 2 independent authors (MK, HA), and any disagreements were resolved by discussing and consulting with a third author (KhRK) to reach a consensus. The quality score ranged from 0 to 5, with those between 0 to 2 indicating low quality, 3 representing medium quality, and those between 4 to 5 reflecting high quality.^[11]^

### 2.6. Synthesis method

Due to the heterogeneity of the interventions and outcome measures, a narrative synthesis was conducted. The findings were summarized in tables, highlighting the characteristics and results of each study.

## 3. Results

**Figure 1.**
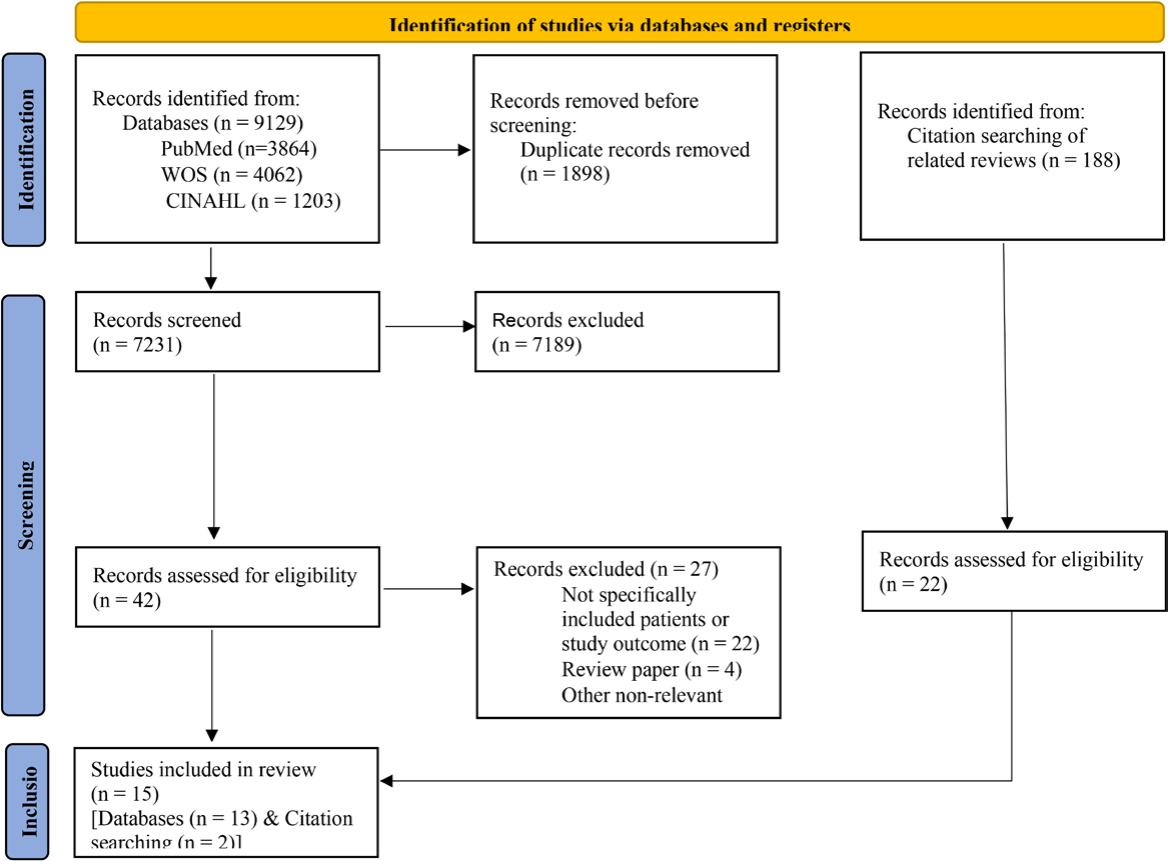
PRISMA 2020: flow diagram of searching, screening, and selecting the records. PRISMA = preferred reporting items for systematic reviews and meta-analyses, WOS = web of science.

The process of searching, screening, and selecting studies for this review is shown in Figure 1. Initially, 9129 records were identified through electronic database searches. After removing duplicates and excluding records based on title and abstract screening, the full texts of 42 studies were assessed. Finally, 15 studies were included in this review as a result of searching in databases and reviewing the reference list of related reviews.^[12-14]^

The characteristics of the included studies are listed in Table 1. According to Table 1, the included trials were performed between 2000 and 2023 in different countries. In addition, the maternal stress was measured using different scales. Various interventions were performed on women during the postpartum period. The duration of the intervention and follow-up varied across the studies. The most common non-pharmacologic interventions were:

**Table 1:** Characteristics of the studies included in the systematic review.

Author (yr)	Country	Study design	Study population	Intervention	Comparison	Outcome (measure)	Duration of intervention/follow-up	Key findings	Study limitation(s)
Chen et al^[15]^	Taiwan	Randomized, controlled trial	Postnatally distressed women aged over 18 yr (n = 60)	Support group (4 weekly meeting of transition to motherhood, postnatal stress management, communication skills, life planning; n = 30)	No intervention (n = 30)	Postnatal stress (perceived stress scale) at baseline and at the end of 4th wk	4 wk	Subjects attended the support sessions had significantly decreased stress	Lack of long-term follow-up
Fraser et al^[16]^	Australia	Randomized, controlled trial	Women with newborns (n = 181)	Home visiting program (n = 90)	No intervention (n = 91)	Parenting stress (parenting stress index) at baseline and 12-mo follow-up	Weekly visit, minimum 18 visit per family	Follow-up evaluation did not demonstrate a positive impact on parenting stress	Not mentioned
Kaaresen et al^[17]^	Norway	Randomized, controlled trial	Mothers giving birth to infants with a birth weight below 2000 g and without congenital anomalies (n = 140)	Modified version of the mother-infant transaction program, including 8 sessions shortly before discharge and 4 home visits by specially trained nurses (n = 71)	No intervention (n = 69)	Parenting stress (parenting stress index) at 6 and 12 months follow-up	90 d after discharge	Mothers in the intervention group reported significantly lower total stress score at 12 mo	Limited number of extremely preterm infants
Tseng et al^[18]^	Taiwan	Randomized, controlled trial	Postnatal women aged at least 18 yr old with mature and normal infant (n = 77)	Listening to music (at home) for at least 30 min a day over 2 wk and receiving regular postpartum care (n = 37)	Regular postpartum care (n = 40)	Postpartum stress (the perceived stress scale) at baseline and 2 wk later	2 wk	There were no significant differences between the 2 groups in terms of the posttest levels of perceived stress	Not mentioned
Ravn et al^[19]^	Norway	Randomized, controlled trial	Mothers/preterm infants (30–36 wk), able to speak, read and write Norwegian, without history of drug/alcohol abuse or severe psychiatric disorders, and with normal infants (n = 106)	Mother–infant Transaction program, including eleven-session 1-h standardized intervention program (n = 56)	No intervention (n = 50)	Maternal stress (Norwegian translation of the parenting stress index) at 6 mo	3 mo	At 6 mo, there was no differences in PSI scores between the mothers in the intervention and control groups on the subscales or total stress	Limited generalizability due to inclusion of Norwegian mothers, multiple testing and relatively small sample size
Hannan et al^[20]^	United States (US)	Randomized, controlled trial	Low income first time mothers 18 yr or older, in good health, who delivered a singleton full term healthy infant (n = 139)	Routine hospital discharge care + advanced practice nurses` follow up telephone calls for week 8 postdischarge (n = 70)	Routine hospital discharge care (n = 69)	Perceived maternal stress (perceived stress scale) at 2 mo	8-wk/2-mo follow-up	The intervention group perceived lower stress than did the control group	Not mentioned
Kudo et al^[21]^	Japan	Non-randomized controlled trial	Healthy mothers experienced vaginal deliveries of a single infant, without any medical complications who agreed to participate in the study (n = 55)	Heart rate variability biofeedback intervention (n = 25)	Not using biofeedback intervention (n = 30)	Psychological stress (Edinburgh postnatal depression scale) on day 4 postpartum and at 1 mo postpartum	1 month	There was a significant decrease in the score of Edinburgh postnatal depression scale in biofeedback group	No random allocation and failure to record resting heart rate variability by electrocardiography
Osman et al^[22]^	Lebanon	Randomized, controlled trial	Healthy first-time mothers with healthy newborns, without multiple or complicated gestation, chronic disease or a baby requiring neonatal intensive care (n = 452)	A 20-min film addressing common stressors in the postpartum period (n = 127) or a 24-h telephone support hotline (n = 121) or film + telephone support hotline (n = 101)	A music CD (n = 103)	Levels of stress (Cohen perceived stress scale [PSS-10]) at 2–3 mo postpartum	8–12 wk postpartum	Both postpartum support film and the 24-h telephone hotline service reduced stress in the postpartum period in first-time mothers	Lack of long-term effect and lack of outcome assessor blindness
Giallo et al^[23]^	Australia	Randomized, controlled trial	18-yr-old or older mothers able to speak English with infants aged 0–7 mo (n = 202)	Professionally-led telephone support (n = 63) or self-directed written support (n = 67)	Waitlist control (n = 72)	Level of stress (depression, anxiety and stress scale-21 [DASS-21])	2- and 6-wk post intervention follow-up	Mothers in the professionally-led intervention reported fewer stress than mothers in the other conditions at post intervention	Limited generalizability due to eligibility criteria, no information to compare the mothers participating and not participating in the study
Navidian et al^[24]^	Iran	Randomized, controlled trial	Women with singleton pregnancy, at full 37–42 wk of pregnancy, without a history of known mental illnesses or substance abuse (n = 100)	Home-based supportive-educational counselling (n = 50)	Usual postpartum care at days 10–15, and 42–45 after delivery by staff at health centres (n = 50)	Postpartum stress (hung postpartum stress scale [HPSS]) 6 wk after delivery	Six weeks after delivery	Home-based supportive-educational intervention had a significant effect on postpartum stress reduction	Due to the hybrid nature of the intervention, it is unclear which part was more or less effective
Yang and Chen^[25]^	Taiwan	Single-blinded, randomized controlled trial	Postnatal women (n = 140)	Aerobic gymnastic exercise at least 3 times (15 min/section) a week for 3 mo using compact disc at home (n = 70)	No intervention (n = 70)	Postpartum stress (perceived stress scale)	3 mo	Participants doing aerobic gymnastic exercise improved significantly in PSS after 4- and 12-wk posttests	Single centre study, limited generalizability, unconcealed randomization, and self-reported measurement of study outcomes
Lewis et al^[26]^	United States (US)	Randomized controlled single blind trial	Postpartum women (4.35–11 wk postpartum) who had a history of depression (n = 450)	Telephone-based exercise intervention (n = 150), or telephone-based wellness/support intervention (n = 150)	Usual care (n = 150)	Perceived stress (perceived stress scale [PSS-14])	6 mo	Exercise interventions may have a protective effect on perceived stress among women at risk of postpartum depression	Limited representativeness of the sample due to advertisement
Mannocci et al^[27]^	Italy	Randomized controlled trial	Women delivering at or beyond term, aged >18 yr old and with normal APGAR scores of the infant (n = 51)	HAPPY MAMA project, home midwifery assistance (n = 23)	No intervention (n = 28)	Stress level (parental stress scale) at baseline, 1 wk after delivery, and after the intervention (1, 3, and 6 mo after delivery)	1 mo after birth, following up of 1 wk, 1, 3, 6 mo after delivery	The stress scale score reduced significantly at the end of the follow-ups	Small sample size, different distribution of maternal education level, different supports during the pregnancy
Cooijmans et al^[28]^	Netherland	Randomized controlled trial	Mothers aged ≥18, with singleton pregnancy, having no history of drug/medication use, no severe health problems, having Dutch language proficiency, and no concurrent participation in other studies (n = 127)	Daily hour of mother-infant skin-to-skin contact during the first 5 postnatal weeks (n = 64)	Care-as-usual (n = 63)	Maternal stress (everyday problems list) at postnatal week 2, 5, 12, and 52	A 5-wk daily hour intervention	Daily skin-to-skin contact, beyond usual care, does not reduce maternal stress	Low protocol adherence, small sample size, and limited generalizability
Guevara et al^[29]^	United States (US)	Randomized controlled trial	Mothers who were >18 yr of age, screened positive for minor or moderate depressive symptoms at their child’s well visit, could read and write in English, and had access to a smartphone or computer tablet (n = 75)	A social media-based parenting program developed for new mothers with mild to moderate depressive symptoms (n = 38)	No intervention (n = 37)	Maternal stress (using the parenting stress index-short form) at baseline and at follow-up 3 mo later	8 weekly postpartum	There was no significant group time interactions for the parenting stress index-short form	Single centre study, and limited generalizability

- Support group.^[15]^
- Home visiting program.^[16]^
- Mother-infant transaction program.^[17,19]^
- Listening to music.^[18]^
- Advanced practice nurses’ follow up through telephone calls.^[20]^
- Heart rate variability biofeedback intervention.^[21]^
- A 20-minute film addressing common stressors in the postpartum period or a 24-hour telephone support hotline.^[22]^
- Professionally-led telephone support or self-directed written support.^[23]^
- Home-based supportive-educational counseling.^[24]^
- Aerobic gymnastic exercise.^[25]^
- Telephone-based exercise intervention or telephone-based wellness/support intervention.^[26]^
- Home midwifery assistance.^[27]^
- Mother-infant skin-to-skin contact.^[28]^
- Social media-based parenting program.^[29]^

Despite the heterogeneity of the interventions and measurement tools across the included studies, the majority (n = 10) indicated the positive effect of postpartum interventions on maternal stress in the postpartum period, as opposed to the other 5 studies.^[16,18,19,28,29]^ Small-scale trials and limited generalizability were the most common study limitations (Table 1). The methodological quality of the included studies is shown in Table 2. As shown in this table, 5 studies had low quality, 3 had medium quality, and 7 had high quality.

**Table 2:** Methodologic quality of the included studies using JADAD scale

Author (yr)	Randomization (yes/no)	Randomization (method)	Blinding (yes/no)	Blinding (method)	An account of all patients	Total score	Qualitative rating
Chen et al^[15]^	1	0	0	0	1	2	Low
Fraser et al^[16]^	1	1	1	1	1	5	High
Kaaresen et al^[17]^	1	1	1	1	1	5	High
Tseng et al^[18]^	1	0	0	0	1	2	Low
Ravn et al^[19]^	1	1	1	0	1	4	High
Hannan^[20]^	1	0	0	0	1	2	Low
Kudo et al^[21]^	0	0	0	0	1	1	Low
Osman et al^[22]^	1	0	1	1	1	4	High
Giallo et al^[23]^	1	1	1	1	1	5	High
Navidian et al^[24]^	1	0	0	0	1	2	Low
Yang and Chen^[25]^	1	0	1	0	1	3	Medium
Lewis et al^[26]^	1	1	1	1	1	5	High
Mannocci et al^[27]^	1	1	0	0	1	3	Medium
Cooijmans et al^[28]^	1	1	0	0	1	3	Medium
Guevara et al^[29]^	1	1	1	1	1	5	High

## 4. Discussion

This systematic review analyzed the effectiveness of non-pharmacological interventions in reducing postpartum maternal stress. The findings indicated that a substantial portion of the included studies demonstrated significant positive effects. Support groups were one of the effective interventions, as they provided a platform for shared experiences and peer support. Participants reported feeling less isolated, which contributed to reduced stress levels.^[1]^ This is consistent with previous literature emphasizing the role of social support in mitigating postpartum stress. Improved maternal mental health correlated with better bonding and attachment, which is crucial for infant development.^[30]^ Despite these promising results, several limitations were noted. Many studies had small sample sizes, limiting the generalizability of their findings.^[4]^ The short duration of follow-up in many studies restricted assessment of long-term effects.^[31]^ Another limitation was the studies’ reliance on self-reported measures, which can introduce bias.^[32]^

Maternal stress levels can significantly differ based on whether an infant is born term or preterm. Preterm birth often poses unique challenges, including prolonged hospital stays, developmental concerns, and increased caregiving demands, which elevate maternal stress. Research indicates that mothers of preterm infants experience higher levels of anxiety and stress compared to mothers of term infants due to the complexities of neonatal intensive care and potential long-term health issues.^[33,34]^ Support groups also play a vital role, offering mothers of preterm infants a platform to share experiences and build a community of understanding. Studies have shown that these groups significantly alleviate feelings of isolation and stress, fostering resilience among participants.^[1]^ The sense of camaraderie and shared experience can be particularly comforting for mothers navigating the challenges of preterm birth.

Additionally, interventions focusing on enhancing mother-infant interactions, such as the Parent Baby Interaction Program, have shown positive outcomes in reducing parenting stress in mothers of preterm infants. These programs emphasize the importance of bonding and attachment, which is crucial for the developmental progress of preterm infants, and help mothers feel more confident and supported in their caregiving roles.^[18,35]^ Evidence suggests that improved mother-infant interactions are linked to better developmental outcomes for preterm infants, highlighting the dual benefits of these interventions for both mother and child.^[30]^

In contrast, mothers of term infants may benefit from interventions that address general postpartum stressors, such as adapting to new routines and managing the physical and emotional changes of the postpartum period. Mindfulness practices, for instance, promote relaxation and present-moment awareness, helping mothers manage the daily challenges of caring for a newborn. These interventions have been shown to reduce stress and enhance maternal well-being, contributing to a more positive postpartum experience.^[2]^ Generally, non-pharmacological interventions offer flexible, personalized support that addresses the unique needs of mothers, whether they have term or preterm infants. These interventions not only alleviate maternal stress but also enhance mother-infant bonding, which will lead to better developmental outcomes for infants. Future research should continue to explore the differential impact of these interventions on mothers of term versus preterm infants, ensuring tailored approaches that optimize maternal and infant health.

Digital delivery of interventions, such as online cognitive behavioral therapy programs, showed promise in expanding access to psychological support. This aligns with the growing body of literature supporting the use of digital health interventions.^[36]^ Online platforms effectively reduce barriers to accessing mental health care, making them viable options for postpartum women who may face logistical challenges.^[37]^ The rise of digital health interventions offers a promising avenue for managing postpartum stress. Online platforms provide flexibility, allowing postpartum women to engage in therapy at their convenience.^[38]^ These interventions effectively reduce stress and depression symptoms, similar to traditional in-person therapy.^[39]^ However, challenges such as access to technology and digital literacy may limit the reach of these programs. While this review highlights the benefits of various interventions, it is important to consider studies with differing results. Not all psychological interventions lead to significant improvements in postpartum stress and depression since there is variability in individual responses.^[40]^ Similarly, some interventions did not demonstrate statistically significant benefits over control groups.^[41]^

Overall, the review underscores the importance of integrating non-pharmacological interventions into standard postpartum care. These interventions offer a safe and effective alternative to pharmacological treatments, particularly for breastfeeding mothers concerned about medication transfer to infants. Further research with larger sample sizes and longer follow-up durations is needed to confirm these findings and inform best practices in postpartum care.

## 5. Conclusion

Despite the heterogeneity found between included studies, non-pharmacological interventions significantly reduce postpartum stress, which may offer safe alternatives to pharmacological treatments, particularly for breastfeeding mothers. However, further research with larger sample sizes, longer follow-up times, and more rigorous methodology is needed to confirm these findings and inform best practices in postpartum care. By prioritizing the mental health of mothers, healthcare providers can significantly enhance both maternal and infant outcomes, contributing to healthier families and communities.

## Acknowledgments

The authors would like to thank the researchers who worked on this issue, making this review possible through their valuable research. It should be noted that the authors used artificial intelligence available from https://www.perplexity.ai/ for English editing of the current article.

## Author contributions

**Conceptualization:** Khadije Rezaie Keikhaie, Maryam Nakhaee Moghadam, Maryam Koochakzai.

**Data curation:** Maryam Nakhaee Moghadam, Maryam Koochakzai.

**Investigation:** Hayedeh Arbabi.

**Methodology:** Hayedeh Arbabi.

**Supervision:** Khadije Rezaie Keikhaie.

**Writing – original draft:** Khadije Rezaie Keikhaie, Maryam Koochakzai.

**Writing – review & editing:** Shahla Faal Siahkal.

## Supplementary Material
